# Microwave Assisted Reactions of Some Azaheterocylic Compounds

**DOI:** 10.3390/molecules14010403

**Published:** 2009-01-15

**Authors:** Gheorghita Zbancioc, Vasilichia Bejan, Marian Risca, Costel Moldoveanu, Ionel I. Mangalagiu

**Affiliations:** “Al. I. Cuza” University of Iasi, Organic Chemistry Department, Bd. Carol 11, 700506 Iasi, Romania

**Keywords:** Microwave *N*-alkylation, Imidazole, Pyrimidine, Pyridazine, Phthalazine

## Abstract

A fast, general, environmentally friendly and facile method for preparation of five- and six-membered ring diazaheterocylic salts under microwave irradiation is presented. The *N*-alkylation reactions of imidazole, pyrimidine, pyridazine and phthalazine have been studied. The microwaves remarkably accelerated these *N*-alkylations, the reaction times decreased dramatically, the reaction conditions were milder, the consumed energy decreased considerably and the amount of solvents used was reduced substantially. Consequently, the microwave assisted alkylation of *N*-containing heterocycles could be considered eco-friendly. In some cases, under MW irradiation the yields are also higher. A comparative study of microwave *vs.* classical conditions (liquid solvents) has been done. Twelve new diazaheterocylic salts of potential practical interest were obtained.

## Introduction

During the last decades microwave (MW) irradiation has became an increasingly valuable tool in organic chemistry, since it is a versatile and facile technique applicable to a large variety of syntheses [1,2,3,4,5,6,7,8,9,10,18]. Thus, a large number of organic reactions can be carried out under MW irradiation to often give higher yields than conventional methods after shorter reaction times and under milder conditions. Furthermore, reactions under MW have the great advantage of using small amounts or no organic solvents (‘solvent free’), thus such reactions are more environmentally friendly and generate less side products. There have been several reports on the successful application of microwave (MW) activation to the alkylation of *N*-containing heterocycles [1,3,4,5,6,7,8,9,10]. Among the latter imidazole, pyrimidine, pyridazine and phthalazine compounds are well known biologically active and medicinally potent anticancer [11,12,13,14], anti-HIV [15,16], antibacterial and antifungal [11,17,18], and antihypertensive agents [11,19]. Moreover, imidazolium salts are potent room temperature ionic liquids of current great interest in industry [20,21].

The aim of this work was to develop a new, efficient and general method for preparation of diazaheterocyle salts derived from imidazole, pyrimidine, pyridazine and phthalazine using MW irradiation. 

## Results and Discussion

In order to obtain the desired azaheterocylic salts, we performed the alkylation of some *N*-heterocyles with five- (imidazole derivatives) and six-membered rings (pyrimidine, pyridazine and phthalazine), under both classical heating and MW irradiation conditions.

**Scheme 1:**
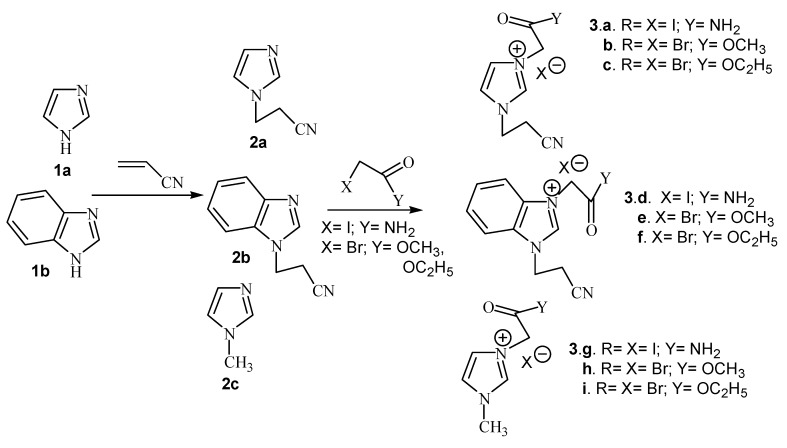
Reaction pathway for the synthesis of imidazolium salts.

Imidazolium salts were obtained in two steps. First we carried out the *N*-cyanoethylation of the acidic nitrogen of imidazole derivatives (imidazole and benzimidazole) via Michael addition of acrylonitrile. In the second step we carried out the quaternization of the second nitrogen atom with iodoacetamide and methyl- or ethyl bromoacetate, respectively (Scheme 1).

**Scheme 2:**
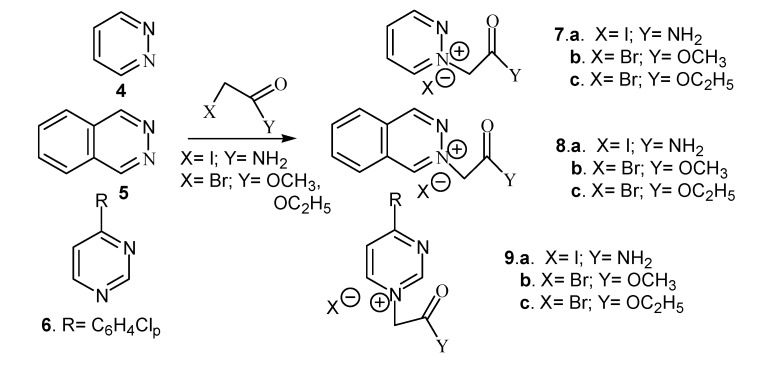
Reaction pathway for synthesis of diazinium salts.

We also performed the quaternization of methyl imidazole with iodoacetamide, methyl- or ethyl bromoacetate, respectively. Diazinium salts were also obtained *via* the quaternization reaction of pyrimidine, pyridazine and phthalazine with the same reagents (Scheme 2).

Under classical heating conditions, these reactions have some major disadvantages, including long reaction times (3-41 h), high energy consumption and the need for large amounts of solvents, etc. For this reason we decided to use microwave technology, a nonconventional method, for the syntheses. The MW assisted reactions were carried out using a monomode reactor, under constant irradiation power and by varying the temperature (the so-called “power control”). The best results were obtained when we used 30% (for imidazole derivatives) and 20% (for diazine derivatives) of the full power of the magnetron (800 W), respectively. Table 1 lists the optimized conditions we employed, under MW irradiation as well as under classical heating. 

**Table 1:** Syntheses of azaheterocyle salts under MW and classical heating conditions, in liquid phase.

Compound	Classical			Microwaves		
	Reaction time	Reaction temp., ^o^C	Yields, %	Reaction time	Reaction temp., ^o^C	Yields, %
**3a**. X=I;Y= NH_2_	20	56	92	10 min.	56	92
**3b**. X=Br;Y= OCH_3_	20	56	84	10 min.	56	87
**3c**. X=Br;Y= OC_2_H_5_	20	56	92	10 min.	56	94
**3d**. X=I;Y= NH_2_	25	56	60	15 min.	56	82
**3e**. X=Br;Y= OCH_3_	25	56	59	15 min.	56	86
**3f**. X=Br;Y= OC_2_H_5_	25	56	87	15 min.	56	88
**3g**. X=I;Y= NH_2_	20	56	88	10 min.	56	88
**3h**. X=Br;Y= OCH_3_	20	56	70	10 min.	56	87
**3i**. X=Br;Y= OC_2_H_5_	20	56	90	10 min.	56	91
**7a**. X=I;Y= NH_2_	3 h	80	86	5 min.	70	89
**7b**. X=Br;Y= OCH_3_	4 h	80	91	5 min.	70	92
**7c**. X=Br;Y= OC_2_H_5_	3 h	80	94	5 min.	70	94
**8a**. X=I;Y= NH_2_	24 h	r.t.	74	5 min.	65	78
**8b**. X=Br;Y= OCH_3_	32 h	r.t.	89	5 min.	65	89
**8c**.X=Br;Y= OC_2_H_5_	24 h	r.t	83	5 min.	65	86
**9a**. X=I;Y= NH_2_	32 h	112	70	10 min.	80	74
**9b**. X=Br;Y= OCH_3_	41 h	112	68	10 min.	80	81
**9c**. X=Br;Y= OC_2_H_5_	32 h	112	66	10 min.	80	80

A comparative analysis for the data obtained leads to the conclusion that the use of MW resulted in a remarkable acceleration of the reactions, with the reaction times decreasing dramatically, from hours to minutes (5 to 15 min.). Also, the reaction temperature decreased in the most cases by 10 to 30 ^o^C, so consequently, the consumed energy decreased considerably too. Moreover, the amount of solvents used is at least three-fold less (see Experimental), so these reactions may be considered as environmentally friendly. It was also of interest that, in some cases, under MW irradiation the yields are higher, sometimes substantially (by almost 20 %). The structure of the compounds synthesized was proven by elemental (C, H, N) and spectral analysis (IR, ^1^H-NMR, ^13^C-NMR, 2D-COSY, 2D-HETCOR (HMQC), long range 2D-HETCOR (HMBC). All the elemental and spectral data are in accordance with the proposed structures and are presented to the Experimental section.

## Conclusions

We report herein a comparative study of the syntheses of diazaheterocylic salts (with five- and six-membered rings) under microwave irradiation and by classical heating in liquid solvents. A fast, general, environmentally friendly, and facile method for preparation of diazaheterocylic salts under microwave irradiation is presented. The microwave irracdiation provided a remarkable rate of acceleration for *N*-alkylation, and the reaction times decreased dramatically, the reaction conditions were milder, the consumed energy decreased considerably and the amount of solvents used was reduced substantially, and consequently, the microwave assisted alkylation of *N*-containing heterocycles could be considered eco-friendly. In some cases, under MW irradiation the yields are also higher, sometimes substantially (by almost 20 %). Twelve new diazaheterocylic salts – **3a-i** and **9a-c** – of potential practical interest were obtained.

## Experimental

### General

All the reagents and solvents employed were of the best grade available and were used without further purification. Melting points were determined using an Electrothermal apparatus and are uncorrected. The ^1^H- and ^13^C-NMR spectra and two-dimensional 2D-COSY, 2D-HETCOR (HMQC), long range 2D-HETCOR (HMBC) experiments were recorded on a Bruker Avance 400 DRX spectrometer operating at 400/100 MHz. Chemical shifts are given in ppm (δ-scale), coupling constants (*J*) in Hz. For the microwave irradiation we used a 800 W STAR SYSTEM-2 monomode reactor (CEM Corporation). The microanalyses were in satisfactory agreement with the calculated values: C, ± 0.15; H, ± 0.10; N, ± 0.30.

### General procedure for synthesis of imidazolium salts **3** under classical heating

Imidazole derivatives **2** (10 mmol) were dissolved in dry acetone (30 mL). A solution of alkylbromoacetate or iodoacetamide (12 mmol) in dry acetone (10 mL) was added dropwise, under stirring. The reaction mixture was then refluxed on an oil bath for 20 h (imidazole derivatives) or 25 h (benzimidazole). The obtained salt was filtered under vacuum, washed with diethyl ether (5 mL) and then purified by recrystallization from an appropriate solvent.

### General procedure for syntheses of diazine salts **7-9** under classical heating

Diazine derivatives (10 mmol) were dissolved in benzene (2 mL, for **7**), methanol (10 mL, for **8**) or toluene (10 mL, for **9**). A solution of alkylbromoacetate or iodoacetamide (12 mmol), in benzene/methanol/toluene (30 mL) was added dropwise, under stirring. The reaction mixture was then refluxed for the appropriate period of time (Table 1). The obtained salts were filtered off, washed twice with the solvent used (10 mL) and then purified by recrystallization from an appropriate solvent.

### General procedure for syntheses of imidazolium (**3**) and diazine salts (**7-9**) under MW irradiation

*Caution!* It is hazardous to rapidly heat reactions under microwave irradiation. Therefore, caution should be exercised when conducting reactions of this type.

Alkylbromoacetate or iodoacetamide [12 mmol, in 15 mL solvent (acetone for imidazole **2**, benzene for **7**, methanol for **8**, toluene for **9**)] was placed in the reaction vessel (Pyrex glass or quartz; for parallel synthesis both cells of the STAR reactor could be used, in which case the irradiation power of reactor has to be double). Imidazole or diazine derivative (10 mmol) was then added. The tubes are then placed in the microwave cell and heated for the appropriate time (Table 1). Stirring of the reaction mixture is desirable. If a stirring device is not available it can be replaced with nitrogen continuously bubbled into the reaction system. Once the heating cycle is complete, the tube was cooled to ambient temperature, removed from the reactor, and the cycloimmonium salts were filtered off, then processed as indicated under classical heating, above.

*1-(Carbamoylmethyl)-3-(2-cyanoethyl)-3-H-imidazol-1-ium iodide* (**3a**)*.* Orange crystals, mp = 164-166°C; ^1^H-NMR (DMSO-D_6_): 3.26-3.23 (t, *J* = 6.4 Hz, 2H, CH_2_: α-cyano), 4.58-4.55 (t, *J* = 6.4 Hz, 2H, CH_2_: β-cyano), 5.02 (s, 2H, CH_2_: α-C=O), 7.52 (s, 1H, NH, H_b_), 7.77-7.76 (d*,* *J* = 1.6 Hz, 1H, CH: H-5), 7.83 (s, 1H, NH, H_a_), 7.85-7.84 (d*,* *J* = 1.6 Hz, 1H, CH: H-4), 9.23 (s, 1H, CH: H-2); ^13^C-NMR (DMSO-D_6_): 18.68 (C, CH_2_: α-cyano), 44.44 (C, CH_2_: β-cyano), 50.62 (C, CH_2_: α-C=O), 117.59 (CN), 121.67 (C-4), 124.25 (C-5), 137.60 (C-2), 166.44 (C, C=O).

*1-(2-cyanoethyl)-3-(2-methoxy-2-oxoethyl)-1H-imidazol-3-ium bromide* (**3b**)*.* White crystals, mp = 129-130°C; ^1^H-NMR (DMSO-D_6_): 3.34-3.31 (t, *J* = 6.4 Hz, 2H, CH_2_: α-cyano), 3.75 (s, 3H, OCH_3_), 4.67-4.64 (t, *J* = 6.4 Hz, 2H, CH_2_: β-cyano), 5.42 (s, 2H, CH_2_: α-C=O), 7.91 (s*,* 1H, CH: H-4), 8.02 (s*,* 1H, CH: H-5), 9.43 (s, 1H, CH: H-2); ^13^C-NMR (CDCl_3_): 18.69, (C, CH_2_: α-cyano), 44.56 (C, CH_2_: β-cyano), 49.66 (C, CH_2_: α-C=O), 52.81 (C, OCH_3_), 117.57 (CN), 122.17 (C-5), 124.13 (C-4), 137.66 (C-2), 167.13 (C, C=O).

*1-(2-Cyanoethyl)-3-(2-ethoxy-2-oxoethyl)-1H-imidazol-3-ium bromide* (**3c**). Yellow oil; ^1^H-NMR (DMSO-D_6_): 1.23-1.20 (t, *J* = 7.2 Hz, 3H, CH_3_), 3.32-3.29 (t, *J* = 6.4 Hz, 2H, CH_2_: α-cyano), 4.21-4.16 (q, *J* = 7.2 Hz, 2H, OCH_2_), 4.66-4.62 (t, *J* = 6.4 Hz, 2H, CH_2_: β-cyano), 5.38 (s, 2H, CH_2_: α-C=O), 7.91-7.90 (d*,* 1H, *J* = 1.2 Hz, CH: H-5), 8.02-8.01 (d*,* 1H, *J* = 1.2 Hz, CH: H-4), 9.44 (s, 1H, CH: H-2); ^13^C-NMR (DMSO-D_6_): 13.93 (C, CH_3_), 18.79, (C, CH_2_: α-cyano), 44.66 (C, OCH_2_), 49.80 (C, CH_2_: β-cyano), 61.95 (C, CH_2_: α-C=O), 117.66 (CN), 122.19 (C-4), 124.18 (C-5), 137.76 (C-2), 166.67 (C, C=O).

*1-(Carbamoylmethyl)-3-(2-cyanoethyl)-3-H-benzo[d]imidazol-1-ium iodide* (**3d**). Yellow crystals, mp = 167-169°C; ^1^H-NMR (DMSO-D_6_): 3.32-3.29 (t, *J* = 6.4 Hz, 2H, CH_2_: α-cyano), 4.95-4.92 (t, *J* = 6.4 Hz, 2H, CH_2_: β-cyano), 5.34 (s, 2H, CH_2_: α-C=O), 7.75-7.70 (m, 2H, H-5, H-6), 7.96-7.92 (m, 3H, H-4, H-7, NH_b_), 8.20 (s, 1H, NH, H_a_), 9.83 (s, 1H, CH, H-2); ^13^C-NMR (DMSO-D_6_): 18.11 (C, CH_2_: α-cyano), 42.40 (C, CH_2_: β-cyano), 48.38 (C, CH_2_: α-C=O), 113.80 (C-7), 113.84 (C-4), 117.87 (CN), 126.73 (C-6), 126.96 (C-5), 130.49 (C-7a), 131.61 (C-3a), 143.73 (C-2), 166.32 (C, C=O).

*1-(2-cyanoethyl)-3-(2-methoxy-2-oxoethyl)-1H-benzo[d]imidazol-3-ium bromide* (**3e**)*.* White crystals, mp = 161-163°C; ^1^H-NMR (DMSO-D_6_): 3.41-3.38 (t, *J* = 6.8 Hz, 2H, CH_2_: α-cyano), 3.78 (s, 3H, OCH_3_), 5.05-5.02 (t, *J* = 6.8 Hz, 2H, CH_2_: β-cyano), 5.77 (s, 2H, CH_2_: α-C=O), 7.75-7.73 (m, 2H, H-5, H-6), 8.14-8.12 (dd*,* *J* = 4.4 Hz, *J* = 2.4 Hz, 1H, CH: H-7), 8.31-8.28 (dd*,* *J* = 4.0 Hz, *J* = 1.6 Hz, 1H, CH: H-4), 10.08 (s, 1H, CH: H-2); ^13^C-NMR (DMSO-D_6_): 18.14, (C, CH_2_: α-cyano), 42.57 (C, CH_2_: β-cyano), 47.67 (C, CH_2_: α-C=O), 53.92 (C, OCH_3_), 113.99 (C-4), 114.10 (C-7), 117.77 (CN), 126.83 (C-6), 127.02 (C-5), 130.35 (C-7a), 131.29 (C-3a), 143.72 (C-2), 166.86 (C, C=O).

*1-(2-Cyanoethyl)-3-(2-ethoxy-2-oxoethyl)-1H-benzo[d]imidazol-3-ium bromide* (**3f**). White crystals, mp = 157-158°C; ^1^H-NMR (DMSO-D_6_): 1.27-1.24 (t, *J* = 7.2 Hz, 3H, CH_3_), 3.38-3.35 (t, *J* = 6.4 Hz, 2H, CH_2_: α-cyano), 4.26-4.21 (q, *J* = 7.2 Hz, 2H, OCH_2_), 5.02-4.99 (t, *J* = 6.4 Hz, 2H, CH_2_: β-cyano), 5.72 (s, 2H, CH_2_: α-C=O), 7.77-7.71 (m, 2H, H-5, H-6), 8.11-8.09 (dd*,* *J* = 5.2 Hz, *J* = 2.4 Hz, CH: H-7), 8.28-8.26 (dd*,* 1H, *J* = 5.2 Hz, *J* = 2.4 Hz, CH: H-4), 10.00 (s, 1H, CH: H-2); ^13^C-NMR (DMSO-D_6_): 13.94 (C, CH_3_), 18.12, (C, CH_2_: α-cyano), 42.56 (C, OCH_2_), 47.69 (C, CH_2_: β-cyano), 62.03 (C, CH_2_: α-C=O), 113.99 (C-7), 114.08 (C-4), 117.79 (CN), 126.87 (C-6), 127.06 (C-5), 130.40 (C-7a), 131.35 (C-3a), 143.75 (C-2), 166.67 (C, C=O).

*1-Carbamoylmethyl-3-methyl-3-H-imidazol-1-ium iodide* (**3g**). Yellow crystals, mp = 186-188°C; ^1^H-NMR (DMSO-D_6_): 3.90 (s, 3H, CH_3_: NCH_3_), 4.98-4.95 (s, 2H, CH_2_: α-C=O), 7.52 (s, 1H, NH, H_b_), 7.70 (s, 2H, H-4, H-5), 7.83 (s, 1H, NH, H_a_), 9.08 (s, 1H, CH: H-2); ^13^C-NMR (DMSO-D_6_): 35.88 (C, NCH_3_), 50.42 (C, CH_2_: α-C=O), 122.86 (C-5), 123.72 (C-4), 137.62 (C-2), 166.44 (C, C=O).

*1-Methyl-3-(2-methoxy-2-oxoethyl)-1H-imidazol-3-ium bromide* (**3h**). White crystals, mp = 135-136°C; ^1^H-NMR (CDCl_3_): 3.75 (s, 3H, OCH_3_), 3.97 (s, 3H, NCH_3_), 5.42 (s, 2H, CH_2_: α-C=O), 7.87-7.86 (m*,* 2H, H-4, H-5), 9.33 (s, 1H, CH: H-2); ^13^C-NMR (CDCl_3_, δ): 35.97 (C, NCH_3_), 49.42 (C, CH_2_: α-C=O), 52.78 (C, OCH_3_), 123.28 (C-5), 123.55 (C-4), 137.54 (C-2), 167.29 (C, C=O).

*1-Methyl-3-(2-ethoxy-2-oxoethyl)-1H-imidazol-3-ium bromide* (**3i**). Yellow oil; ^1^H-NMR (CDCl_3_): 1.32-1.28 (t, *J* = 7.2 Hz, 3H, CH_3_), 4.10 (s, 3H, NCH_3_), 4.28-4.23 (q, *J* = 7.2 Hz, 2H, OCH_2_), 5.47 (s, 2H, CH_2_: α-C=O), 7.75-7.74 (d*,* *J* = 3.2 Hz, CH: H-5), 7.83-7.82 (d*,* *J* = 3.2 Hz, CH: H-4), 9.78 (s, 1H, CH: H-2); ^13^C-NMR (CDCl_3_): 14.12 (C, CH_3_), 36.94 (C, NCH_3_), 50.32 (C, OCH_2_), 62.86 (C, CH_2_: α-C=O), 123.45 (C-5), 124.04 (C-4), 137.84 (C-2), 166.67 (C, C=O).

*1-Ethoxycarbonylmethyl-pyridazin-1-ium bromide* (**7c**). White crystals, mp = 160-161°C; ^1^H-NMR (CDCl_3_): 1.36-1.33 (t, *J* = 7.2 Hz, 3H, CH_3_), 4.36-4.30 (q, *J* = 7.2 Hz, 2H, OCH_2_), 6.19 (s, 2H, CH_2_: α-C=O), 8.65 (m*,* 1H, CH: H-4), 8.96 (m, 1H, CH: H-5), 9.44-9.43 (d, *J* = 6.0 Hz, 1H, CH: H-3), 10.96-10.95 (d, *J* = 6.0 Hz, 1H, CH: H-6); ^13^C-NMR (CDCl_3_): 14.04 (C, CH_3_), 63.67 (C, OCH_2_), 65.30 (C, CH_2_: α-C=O), 136.39 (C-5), 137.71 (C-4), 151.96 (C-6), 153.96 (C-3), 164.34 (C, C=O).

*2-Ethoxycarbonylmethyl-phthalazin-2-ium bromide* (**8c**). Yellow crystals, mp = 144-145°C; ^1^H-NMR (CDCl_3_): 1.37-1.34 (t, *J* = 7.2 Hz, 3H, CH_3_), 4.37-4.32 (q, *J* = 7.2 Hz, 2H, OCH_2_), 6.04 (s, 2H, CH_2_: α-C=O), 8.39-8.35 (td, *J* = 8.4 Hz, *J* = 1.2 Hz, 1H, CH: H-6), 8.50-8.46 (td, *J* = 8.4 Hz, *J* = 1.2 Hz, 1H, CH: H-7), 8.60-8.58 (d*,* *J* = 7.6 Hz, 1H, CH: H-5), 8.85-8.83 (d*,* *J* = 7.6 Hz, 1H, CH: H-8), 9.88 (s, 1H, CH: H-4), 11.78 (s, 1H, CH: H-1); ^13^C-NMR (CDCl_3_): 14.02 (C, CH_3_), 63.49 (C, CH_2_: α-C=O), 63.66 (CH_2_), 127.39 (C-8a), 128.04 (C-4a), 128.20 (C-8), 131.43 (C-7), 136.54 (C-5), 140.07 (C-4), 153.85 (C-1), 164.80 (C, C=O).

*1-Carbamoylmetyl-4-(4-chlorophenyl)-pyrimidin-1-ium iodide* (**9a**). Yellow crystals, mp = 123-125°C; ^1^H-NMR (CDCl_3_): 5.10-5.07 (s, 2H, CH_2_: α-C=O), 7.52-7.50 (d, *J* = 8.4 Hz, 2H, 2CH: H-3’), 7.85 (s, 1H, NH_b_), 8.09 (s, 1H, NH_a_), 8.32-8.30 (d, *J* = 8.4 Hz, 2H, 2CH: H-2’), 8.76-8.74 (d, *J* = 6.0 Hz, 1H, CH: H-5), 9.77-9.76 (d, *J* = 6.0 Hz, 1H, CH: H-6), 9.91 (s, 1H, CH: H-2); ^13^C-NMR (CDCl_3_): 56.49 (CH_2_), 118.04 (C-5), 130.26 (C-2’), 130.57 (C-4’), 130.98 (C-3’), 131.32 (C-1’), 153.15 (C-6), 153.83 (C-2), 167.72 (C-4), 167.78 (C, C=O).

*4-(4-Chlorophenyl)-1-methoxycarbonylmethyl-pyrimidin-1-ium bromide* (**9b**). Pink crystals, mp = 102-104°C; ^1^H-NMR (CDCl_3_): 3.78 (s, 3H, CH_3_), 6.18 (s, 2H, CH_2_: α-C=O), 7.46-7.44 (d, *J* = 8.4 Hz, 2H, 2CH: H-3’), 8.25-8.23 (d, *J* = 8.4 Hz, 2H, 2CH: H-2’), 8.74-8.72 (d, *J* = 6.0 Hz, 1H, CH: H-5), 9.75-9.74 (d, *J* = 6.0 Hz, 1H, CH: H-6), 9.89 (s, 1H, CH: H-2); ^13^C-NMR (CDCl_3_): 54.05 (C, CH_3_), 57.73 (CH_2_), 118.11 (C-5), 130.28 (C-2’), 130.60 (C-4’), 131.01 (C-3’), 131.34 (C-1’), 153.16 (C-6), 153.85 (C-2), 167.74 (C-4), 168.83 (C, C=O).

*4-(4-Chlorophenyl)-1-ethoxycarbonylmethyl-pyrimidin-1-ium bromide* (**9c**). Pink crystals, mp = 114-116°C; ^1^H-NMR (CDCl_3_): 1.36-1.33 (t, *J* = 7.2 Hz, 3H, CH_3_), 4.36-4.31 (q, *J* = 7.2 Hz, 2H, OCH_2_), 6.19 (s, 2H, CH_2_: α-C=O), 7.48-7.46 (d, *J* = 8.4 Hz, 2H, 2CH: H-3’), 8.26-8.24 (d, *J* = 8.4 Hz, 2H, 2CH: H-2’), 8.76-8.74 (d, *J* = 6.0 Hz, 1H, CH: H-5), 9.75-9.74 (d, *J* = 6.0 Hz, 1H, CH: H-6), 9.90 (s, 1H, CH: H-2); ^13^C-NMR (CDCl_3_): 14.04 (C, CH_3_), 63.51 (C, CH_2_: α-C=O), 57.77 (CH_2_), 118.14 (C-5), 130.34 (C-2’), 130.61 (C-4’), 131.05 (C-3’), 131.37 (C-1’), 153.18 (C-6), 153.86 (C-2), 167.78 (C-4), 168.87 (C, C=O).
